# The Influence of Sagittal Head Tilt on Periorbital Appearance: Implications for Clinical Photography and the Evaluation of Postoperative Results

**DOI:** 10.1093/asjof/ojab043

**Published:** 2021-10-30

**Authors:** Elbert E Vaca, Jonathan T Bricker, Lauren M Mioton, Steven Fagien, Mohammed S Alghoul

## Abstract

**Background:**

Consistency in standardized periorbital photography—specifically, controlling for sagittal head tilt—is challenging yet critical for accurate assessment of preoperative and postoperative images.

**Objectives:**

To systematically assess differences in topographic measurements and perceived periorbital attractiveness at varying degrees of sagittal head tilt.

**Methods:**

Standardized frontal photographs were obtained from 12 female volunteers (mean age 27.5 years) with the Frankfort plane between −15° and +15°. Unilateral periorbital areas were cropped, and topographic measurements were obtained. The images of each individual eye, at varying head tilt, were ranked in order of attractiveness by 11 blinded evaluators.

**Results:**

Inter-rater and intra-rater reliability was excellent (intraclass correlation > 0.9). Downward sagittal head tilt was linearly associated with an improved aesthetic rating (Spearman’s correlation; ρ = 0.901, *P* < 0.001). However, on subgroup analysis, eyes with lower lid bags received the highest aesthetic score at neutral head tilt. Pretarsal show and upper lid fold heights progressively decreased (*P* < 0.001), positive intercanthal tilt became more pronounced (*P* < 0.001), and the apex of the brow (*P* < 0.001) and lid crease (*P* = 0.036) arcs lateralized with downward sagittal head tilt, contributing to a more angular appearance of the eye. Marginal reflex distance (MRD) 1 was maintained, while MRD2 progressively increased (*P* < 0.001) with downward head tilt.

**Conclusions:**

Negative sagittal head tilt significantly improves periorbital aesthetics; however, in the presence of lower eyelid bags, this also increases demarcation of the eyelid cheek junction which may be aesthetically detrimental. Controlling for sagittal head tilt is critical to reliably compare preoperative and postoperative clinical photographs.

Consistency in standardized clinical photography is challenging but essential for patient preoperative assessment and, perhaps more importantly, for critical evaluation and accurate representation of postoperative results. Differences in lighting, expression, and makeup are known to significantly influence facial appearance.^1-8^ Furthermore, facial appearance is affected by head position as neck flexion accentuates the jowls, submental adiposity, and lower facial skin laxity resulting in a less appealing cervicomental angle.^2,9^ However, the influence of head position on periorbital appearance is less well described in the plastic surgery literature.

We have previously demonstrated the importance of the aesthetic relationship between the arcs of the brow, upper lid crease, and lash line—these arcs define the boundaries of the upper lid fold and pretarsal space.^10^ In young attractive females, the peaks of these 3 arcs should progressively lateralize from caudal to cranial; the contour of these arcs should be smooth, resulting in a relatively consistent height of the pretarsal space, while the upper lid fold height increases from the nasal to lateral aspect of the upper eyelid. Furthermore, compared with less attractive eyes, the youthful attractive female eye is characterized by less pretarsal show, greater positive intercanthal tilt, and negative angulation of the medial brow,^10-12^ giving the eye a more angular appearance.

On close observation of female model photographs, we have noticed a tendency for models to tilt their heads downward—we believe that this serves to accentuate upper periorbital aesthetics (**Figure 1**). Downward head tilt results in a more exaggerated positive intercanthal tilt, downward angulation of the medial brow, and decreased pretarsal show. However, we have observed the opposite relationship in the lower lid where downward head tilt results in increased scleral show and demarcation of the eyelid-cheek junction due to shadow effect, particularly if lower lid orbital fat herniation is present.

**Figure 1.**
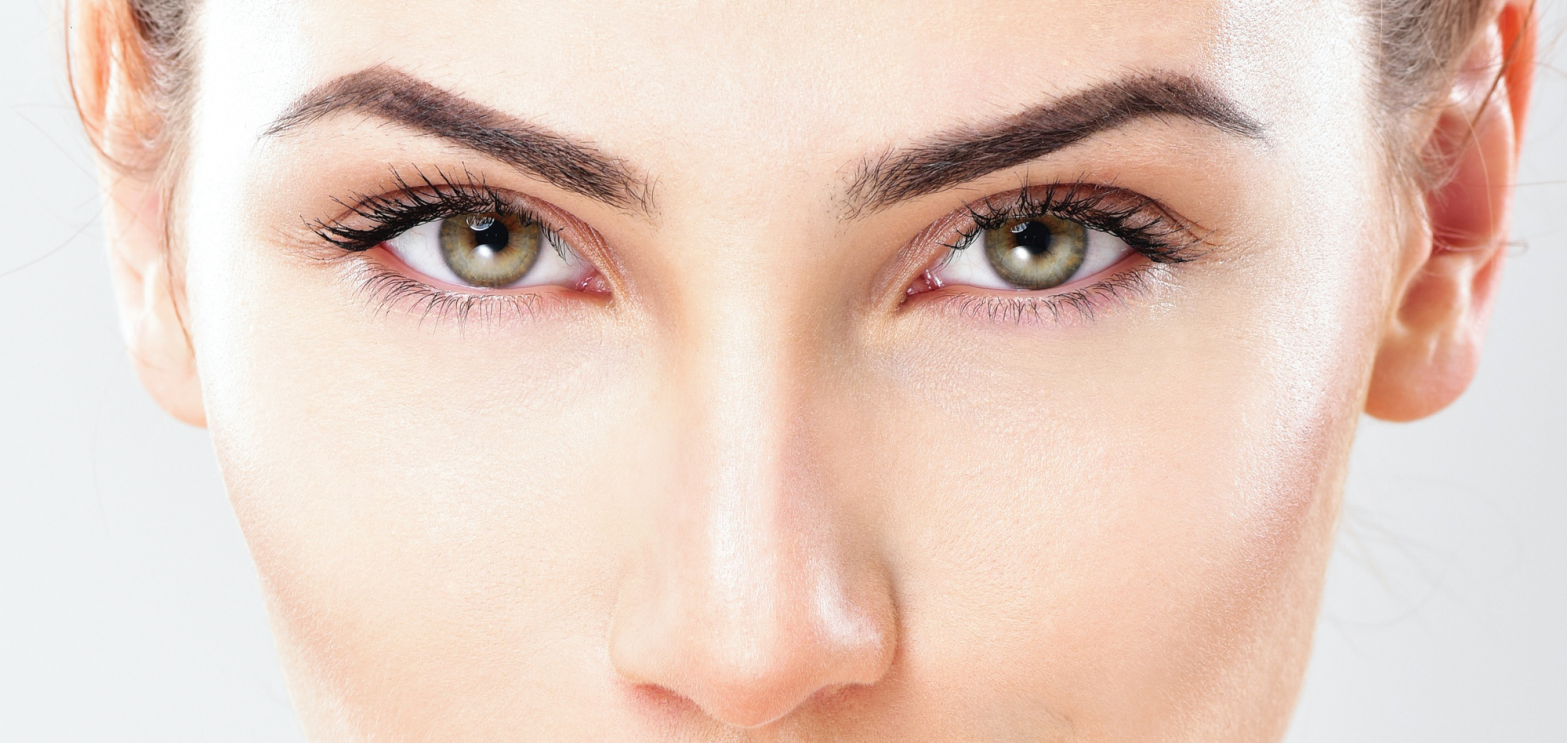
Frontal female model photograph (age unknown, image rights obtained from Shutterstock) demonstrating attractive periorbital features with a positive intercanthal tilt, smooth eyelid cheek-junction transition, and relatively homogeneous height of the pretarsal space from the nasal to lateral aspects of the upper lid. Additionally, note the aesthetically pleasing smooth and youthful arcs of the upper lid crease and brow, with the peak of the lid crease arc located lateral to the midpupil and peak of the brow arc located approximately at the lateral canthus. Importantly, note the subjects ear position—the cephalad margin of the tragus reveals that the subject is tilting her head downward (ie, negative sagittal head tilt). This head position increases the apparent angularity of the eyes and accentuates her positive intercanthal tilt*.*

To our frustration, we have noticed that despite our best attempts to standardize clinical periorbital photographs, there can be subtle differences in sagittal head tilt between preoperative and postoperative photographs, which can significantly affect eyelid aesthetics. To that aim, the goal of this present study is to systematically characterize the influence of sagittal head tilt on periorbital appearance. With these data, we hope to bring attention to the importance of sagittal head tilt when critically analyzing periorbital clinical photographs and postoperative results.

## METHODS

Twelve consecutive female volunteers between the ages of 18 and 40 (mean age 27.5 [range, 22-36]; 10 Caucasian, 2 Hispanic), for a total of 24 eyes, were included in the present study. This study was approved by Northwestern Feinberg School of Medicine’s Internal Review Board, and subject photographs were taken on December 5, 2019, and February 6, 2020, and written consent was obtained from all subjects. 

**Figure 2.**
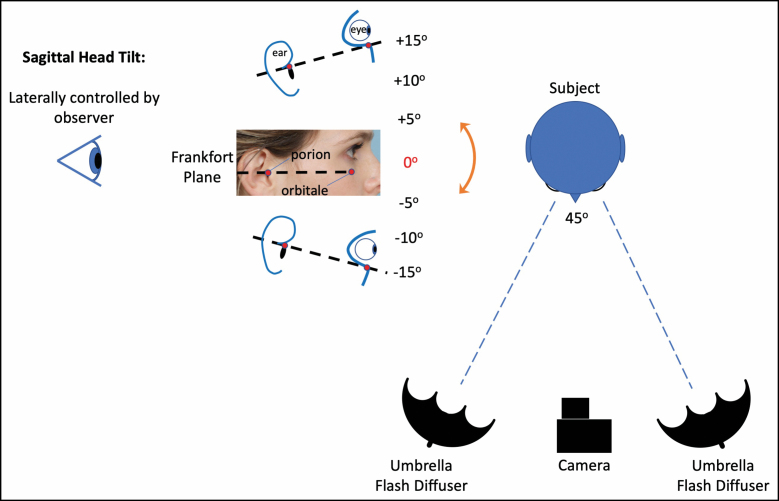
Pictogram demonstrating the standardized photograph acquisition process. An observer was situated lateral to a 28-year-old Caucasian female (90° to the subject’s right) to obtain photographs with the subjects Frankfort plane at 5° increments between −15° and +15°. The camera was placed at eye level directly in front of the subject with a focal length of 80 mm (Video 1). Artwork created by E.E.V.

A Canon EOS 7D DSLR camera with an EF-S 18-135mm lens (Melville, NY) was leveled at the height of the subject’s eyes with an effective focal length of 80 mm. Photographs were taken with the subject’s gaze directed toward the camera lens with neutral facial expression. The Frankfort plane (ie, sagittal head tilt) was controlled by an observer situated 90° lateral to the right of the subject, using the cephalic apex of the external auditory canal (porion) and the soft tissue landmark overlying the most caudal aspect of the inferior orbital rim (orbitale). Photographs were taken of all 12 subjects with the Frankfort plane at 0°, +15°, and −15° with respect to the horizontal plane. Of note, in 5 subjects, additional photographs were taken with the Frankfort plane at −10°, −5°, +5°, and +10°, as detailed in **Figure 2** and Video 1. All photographs were taken in the same sitting; therefore, each patient served as their own internal control for variables such as makeup, weight fluctuations, and daily variations in periorbital edema. 

**Figure 3.**
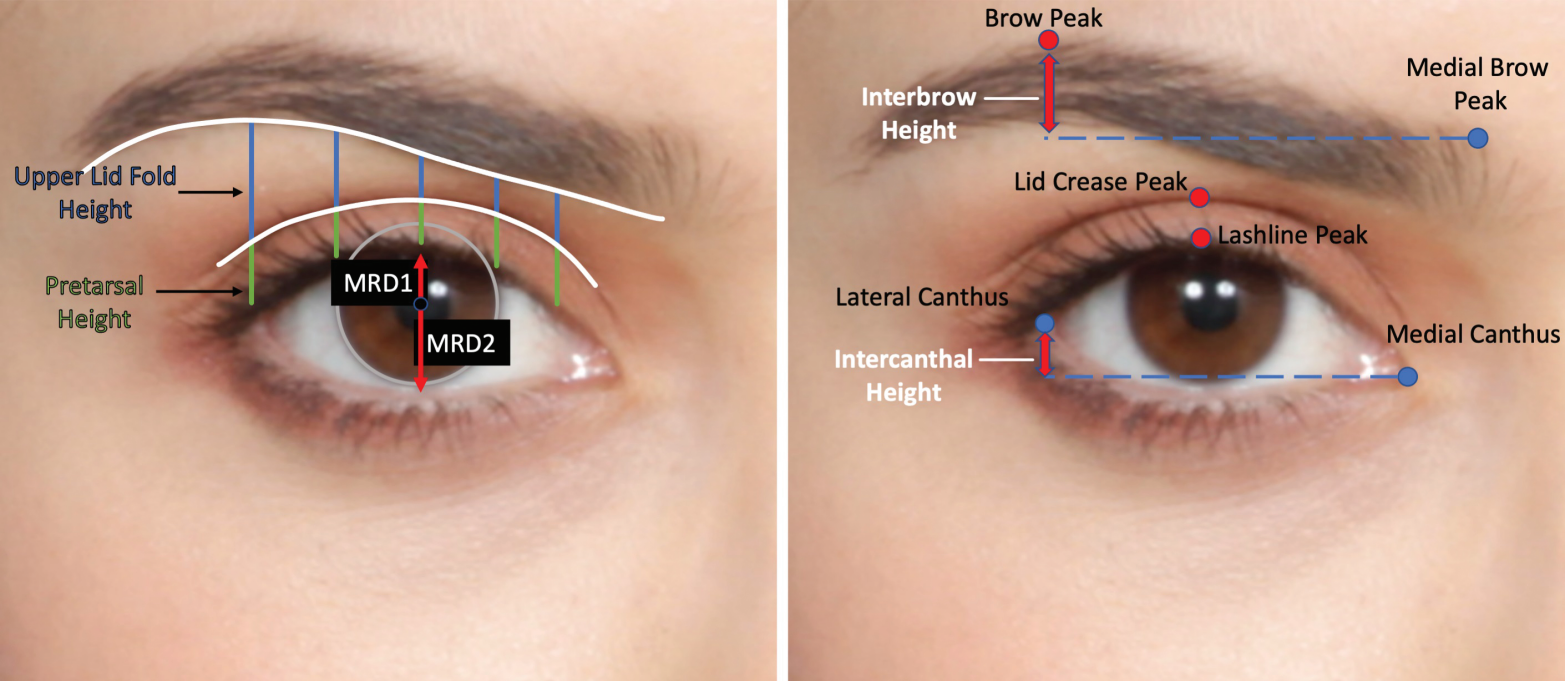
Periorbital topographic analysis in a 23-year-old Hispanic female. (*Left*) The scleral limbus was standardized to a diameter of 11.5 mm (gray circle). The lid crease and inferior brow margins were traced (white), and the vertical heights of the pretarsal space (green) and upper lid fold (blue) were measured at the lateral canthus, lateral limbus, midpupil, medial limbus, and punctum. The upper and lower marginal reflex distances (MRD1 and MRD2, respectively) were measured from the center of the circle corresponding to the scleral limbus to the upper and lower lid margin, respectively. (*Right*) The interbrow height was measured as vertical height discrepancy between the brow peak and medial brow cephalic margin. The intercanthal height was measured as the vertical height discrepancy between the medial and lateral canthus. The lashline peak, lid crease peak, and brow peak were also identified (red circles), and their horizontal location was measured using the midpupil and lateral canthus as a reference point, respectively. Of note, the peak locations were objectively determined utilizing the “measure” tool in Adobe Illustrator by determining where tracings of the brow and lid crease changed from a positive to negative slope (ie, 0-degree tangent).

Each eye was categorized as having a positive or negative globe vector by assessing the anteroposterior relationship between the most projecting aspect of the cornea and malar eminence on lateral view. Eyes were also dichotomized into those with and without readily visible lower lid bags with the Frankfort plane at 0°, by consensus of 2 of this study’s authors. Vertical facial midlines were aligned using the center of the glabella and nadir of cupids bow. Unilateral periorbital areas were cropped, standardized, and topographically analyzed using Adobe Illustrator (San Jose, CA), as described in previous studies by our group (**Figure 3**).^10,13^

Periorbital photographs were rated by 11 evaluators (3 plastic surgeons [2 males, 1 female], 1 physician assistant [female], 2 nurses [female], 2 engineers [male], 1 attorney [female], 1 interior designer [female], and 1 small business owner [male]; 6 Caucasian, 4 Hispanic, and 1 African American; average age 33.5 years) between March 1 and March 30, 2020. Of note, the nose was obscured in all images to minimize potential confounding due to nasal feature alterations. For the ratings, the 3 views of each individual eye (−15°, 0°, and +15°) were placed side by side and randomized in respect to their order from left to right, and thus each eye served as its own internal control with sagittal head tilt as the independent variable. The evaluators were provided the randomized images in a PowerPoint (Microsoft Corp., Redmond, WA) file through a secured email server by J.T.B. and instructed to rank *the overall attractiveness* of the 3 views of each eye from 1 to 3 (1 = most attractive, 3 = least attractive) in a text box located below each eye photograph. Furthermore, for the 5 subjects where 7 sagittal head tilt views were obtained (−15°, −10°, −5°, 0, +5°, +10°, and +15°), evaluators were presented the 7 views of each individual eye side by side and ranked the overall attractiveness of each view (1 = most attractive, 7 = least attractive) after completion of the 3-view survey. A copy of the 3-view and 7-view surveys is available in Appendices A and B.

Statistical comparisons were made between the sagittal head tilt cohorts utilizing SPSS (Armonk, NY). Averages and standard deviations were calculated in millimeters (mm). Mann-Whitney *U* and Kruskal-Wallis tests were used for comparing ordinal data between 2 or more groups, respectively. Pearson’s and Spearman’s correlations were used to assess correlation between 2 continuous variables or a continuous variable and an ordinal variable, respectively. Intraclass correlations (ICC) with a 2-way mixed model assessing consistency or absolute agreement were used to calculate inter-rater reliability and intra-rater reliability, respectively. A *P*-value <0.05 indicated statistical significance.

## RESULTS

Of the 24 analyzed eyes, 16 eyes had a positive and 8 eyes had a negative vector. Four of the negative vector eyes had lower lid bags on 0° head tilt. For the 3-view and 7-view sagittal head tilt attractive ratings, inter-rater reliability (ICC = 0.975 [*P* < 0.001], ICC = 0.961 [*P* < 0.001], respectively) and average intra-rater reliability (ICC = 0.940 [*P* < 0.001], ICC = 0.920 [*P* < 0.001], respectively) were indicative of excellent inter-rater and intra-rater agreement.

### Aesthetic Ratings and Sagittal Head Tilt

**Figure 4.**
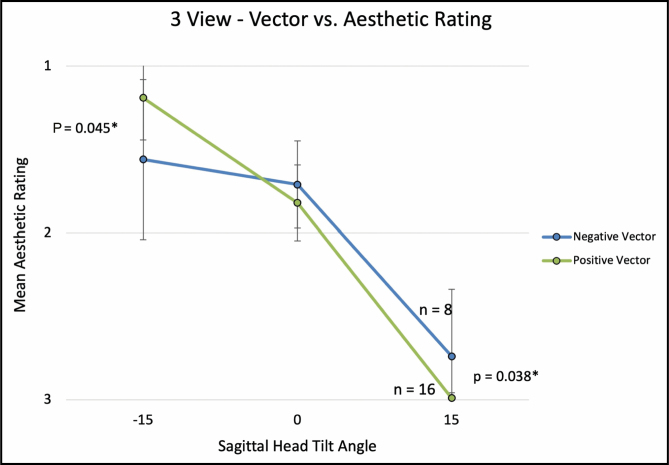
The graph illustrates 3-View, globe vector vs aesthetic rating. Error bars indicate 95% confidence intervals. Asterisk (*) denotes statistically significant values.

**Figure 5.**
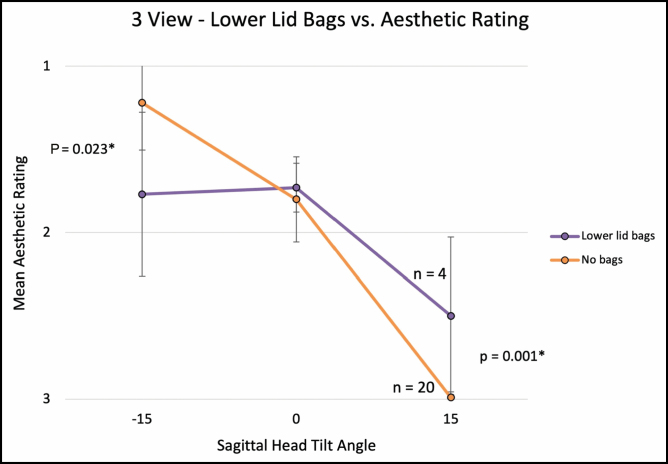
The graph illustrates 3-View, presence of lower eyelid bags vs aesthetic rating. Error bars indicate 95% confidence intervals. Asterisk (*) denotes statistically significant values.

For the 3-view (−15, 0, and +15 degrees) comparison, average aesthetic ratings (1 = most attractive, 3 = least attractive) were 1.31 (±0.38), 1.79 (±0.24), and 2.91 (±0.25), with a significant linear association between a higher aesthetic rating and negative (ie, downward) sagittal head tilt (Spearman’s correlation; ρ = 0.869, *P* < 0.001). Mann-Whitney *U* test revealed that negative sagittal head tilt was significantly associated with worse aesthetic scores in eyes with a negative vector and lower lid bags, with a stronger detrimental effect on aesthetic scores with the presence of lower lid bags (**Figures 4, 5**).

**Figure 6.**
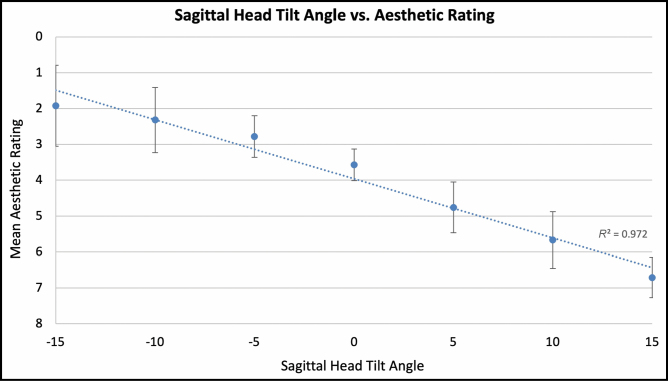
The graph illustrates 7-View, sagittal head tilt vs aesthetic rating. Error bars indicate 95% confidence intervals.

**Figure 7.**
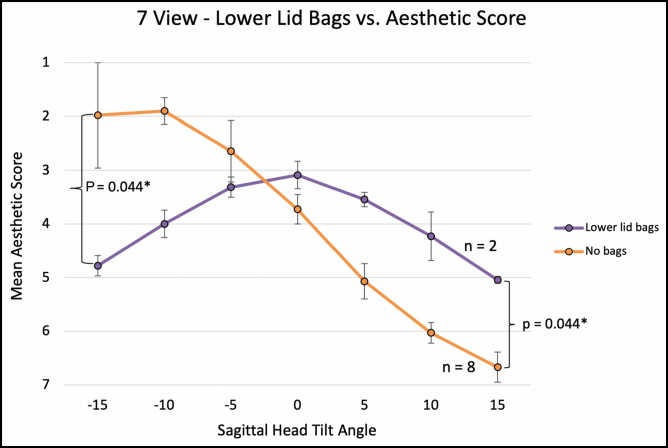
The graph illustrates 7-view, presence of lower lid bags vs aesthetic rating at varying sagittal head tilt. Error bars indicate 95% confidence intervals. Asterisk (*) denotes statistically significant values.

**Figure 8.**
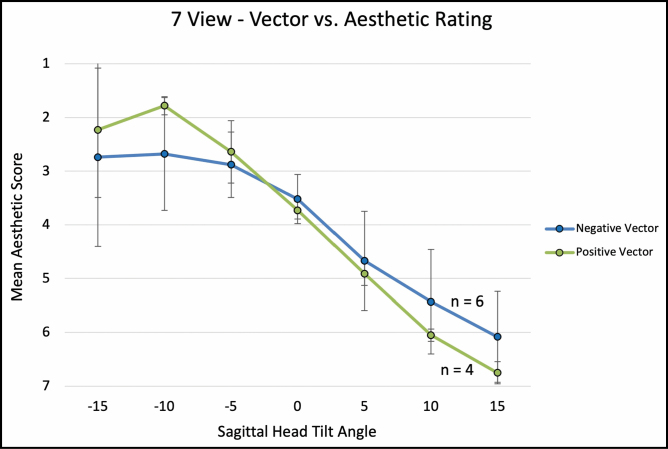
The graph illustrates 7-view, globe vector vs aesthetic rating at varying sagittal head tilt. Error bars indicate 95% confidence intervals. Asterisk (*) denotes statistically significant values.

For the 7-view (−15, −10, −5, 0, +5, +10, and +15 degrees) comparison, average aesthetic ratings (1 = most attractive, 7 = least attractive) were 1.92 (±1.13), 2.32 (±0.91), 2.78 (±0.58), 3.57 (±0.44), 4.76 (±0.71), 5.67 (±0.79), and 6.71 (±0.56), with a significant linear association between a higher aesthetic rating and negative (ie, downward) sagittal head tilt (Spearman’s correlation; ρ = 0.901, *P* < 0.001; **Figure 6**). Ordinal regression revealed that lower eyelid bags were independently associated with worse aesthetic ratings with negative head tilt (*P* = 0.049). Interestingly, when mean aesthetic ratings were compared between eyes with and without lower lid bags across varying degrees of sagittal head tilt, eyes without lower lid bags followed a sigmoidal curve with better aesthetic scores with negative head tilt. Meanwhile, eyelid with lower lid bags followed a parabolic curve with better aesthetic scores with neutral (0°) head tilt (**Figure 7**). For negative vector eyes, there was a nonsignificant trend for worse aesthetic ratings with negative head tilt (**Figure 8**).

### Periorbital Arc Changes, Marginal Reflex Distance, and Scleral Show

Periorbital arc, marginal reflex distance (MRD), and intercanthal height changes with sagittal head tilt are presented in **Table 1**. Lashline, lid crease, and brow arc peak lateralization were correlated with a higher aesthetic rating (*P* < 0.001; **Table 2**).

**Table 1.** Periorbital Arc, Marginal Reflex Distance (MRD), and Scleral Show Changes With Variation in Sagittal Head Tilt

	Sagittal head tilt angle						
	−15	−10	−5	0	+5	+10	+15
Brow peak height (from lateral canthus), mm (mean ± SD)	20.5 ± 2.90	20.7 ± 1.97	22.6 ± 2.00	24.2 ± 2.56	24.9 ± 1.98	25.9 ± 2.05	27.7 ± 1.90
Pearson correlation	0.757						
*P*-value	<0.001*						
Interbrow height, mm (mean ± SD)	9.51 ± 2.01	9.30 ± 2.49	8.83 ± 2.30	7.33 ± 2.08	7.46 ± 2.68	6.31 ± 2.76	4.82 ± 2.01
Pearson correlation^a^	−0.61						
*P* value	<0.001*						
Brow peak HL (from lateral canthus), mm (mean ± SD)	−1.16 ± 2.81	−0.380 ± 2.03	0.626 ± 2.21	2.60 ± 2.30	2.00 ± 2.73	5.94 ± 4.48	8.82 ± 3.46
Pearson correlation	0.759						
*P* value	<0.001*						
Lid crease peak HL (from midpupil), mm (mean ± SD)	−0.326 ± 1.76	0.857 ± 2.52	0.807 ± 2.24	0.0150 ± 1.66	1.47 ± 1.85	1.25 ± 1.49	0.730 ± 1.61
Pearson correlation	0.199						
*P* value	0.036*						
Lashline peak HL (from midpupil), mm (mean ± SD)	−0.841 ± 0.602	−0.153 ± 0.966	−0.256 ± 0.927	−0.249 ± 0.728	0.392 ± 0.783	0.884 ± 1.06	0.589 ± 0.705
Pearson correlation	0.563						
*P* value	<0.001*						
Vertical palpebral aperture (at midpupil), mm (mean ± SD)	10.9 ± 1.40	11.2 ± 1.14	11.1 ± 0.917	10.6 ± 1.05	10.4 ± 0.962	9.72 ± 1.41	8.92 ± 1.31
Pearson correlation^a^	−0.52						
*P* value	<0.001*						
MRD1, mm (mean ± SD)	4.07 ± 0.934	4.06 ± 1.08	4.31 ± 0.905	4.54 ± 0.779	4.27 ± 0.767	4.04 ± 0.863	4.14 ± 0.824
Pearson correlation	0.021						
*P* value	0.826						
MRD2, mm (mean ± SD)	6.74 ± 0.927	7.07 ± 0.389	6.73 ± 0.628	5.93 ± 0.675	6.00 ± 0.641	5.49 ± 0.668	4.66 ± 0.905
Pearson correlation^a^	−0.698						
*P* value	<0.001*						
Scleral show^b^, mm (mean ± SD)	1.10 ± 0.905	0.264 ± 0.668	−0.245 ± 0.641	−0.181 ± 0.675	−0.980 ± 0.628	−1.32 ± 0.389	−0.986 ± 0.927
Pearson correlation^a^	−0.698						
*P* value	<0.001*						
Intercanthal height, mm (mean ± SD)	4.39 ± 1.47	3.79 ± 1.43	3.06 ± 1.07	2.55 ± 1.33	1.82 ± 1.13	1.06 ± 1.55	0.128 ± 1.35
Pearson correlation^a^	−0.757						
*P* value	<0.001*						

Negative value indicates that landmark is lateral to reference point. HL, horizontal location; MRD, marginal reflex distance; SD, standard deviation. ^a^Negative Pearson correlation signifies an increase in variable associated with a decrease in sagittal head tilt. ^b^Scleral show (SS) was calculated as: SS = MRD2 − 5.75 mm (ie, radius of scleral limbus). *Statistically significant.

**Table 2.** Spearman’s Correlation Between Periorbital Arc, Marginal Reflex Distance (MRD) and Scleral Show Changes, and Aesthetic Rating

	Spearman’s correlation^a^	*P* value
Brow peak height (from lateral canthus)	−0.404	<0.001*
Interbrow height	0.23	0.015*
Brow peak HL (from lateral canthus)	−0.434	<0.001*
Lid crease peak HL (from midpupil)	−0.404	<0.001*
Lashline peak HL (from midpupil)	−0.493	<0.001*
Vertical palpebral aperture (at midpupil)	0.155	0.102
MRD1	0.003	0.972
MRD2	−0.248	0.008*
Scleral show	−0.248	0.008*
Intercanthal height	0.479	<0.001*

HL, horizontal location; MRD, marginal reflex distance. ^a^Negative Spearman’s correlation signifies improved aesthetic rating associated with a decrease in the respective variable; note that lateralization of brow, lid crease, and lashline peak location was associated with an improved aesthetic rating. *Statistically significant.

**Figure 9.**
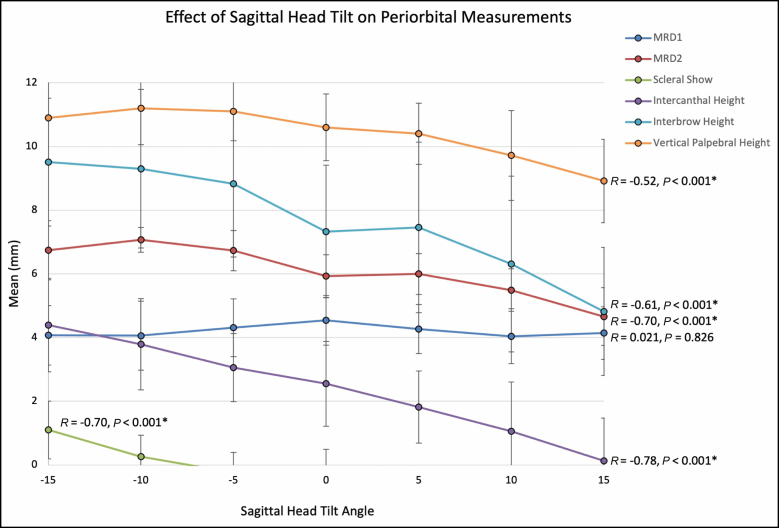
Effect of sagittal head tilt on periorbital measurements. Upper and lower lid marginal reflex distance (MRD1 and MRD2, respectively). Note that intercanthal height, interbrow height, vertical palpebral height (ie, MRD1 + MRD2), MRD2, and scleral show significantly increase with negative sagittal head tilt. There was no significant change in MRD1. Of note, scleral show was calculated using MRD2 – 5.75 mm (radius of scleral limbus). Error bars indicate 95% confidence intervals. Asterisk (*) denotes statistically significant values.

MRD1 did not significantly change across −15° to +15° of sagittal head tilt (*P* = 0.826), while in MRD2, scleral show, intercanthal height, and interbrow height significantly increased with negative head tilt (*P* < 0.001; **Table 1**). An increase in intercanthal height (ie, positive intercanthal tilt) and interbrow height (ie, downward medial brow angulation) was correlated with a higher aesthetic rating (**Table 2**; **Figure 9**).

### Pretarsal and Upper Lid Fold Measurements

Mean pretarsal and upper lid fold heights from the medial to lateral aspects of the lid at varying sagittal head tilt are presented in **Table 3**, demonstrating a decrease in the vertical height of the pretarsal space and upper lid fold (with a decrease in medial and maintenance of ($\frac{Upper\;Lid\;Fold}{Pretarsal}$) ratios at the midpupil) with decreasing sagittal head tilt. Further analysis revealed a correlation between improved aesthetic rating and decreasing height of both the pretarsal space and upper lid fold across the upper lid (Spearman’s correlation, ρ < 0.001; **Table 4**).

**Table 3.** Upper Periorbital Measurement and Ratio Changes With Variation in Sagittal Head Tilt

		Sagittal head tilt angle						
		−15	−10	−5	0	+5	+10	+15
Punctum	Upper lid fold, mm (mean ± SD)	4.00 ± 1.78	3.50 ± 1.11	4.30 ± 1.41	6.51 ± 1.96	5.65 ± 1.38	6.69 ± 1.77	9.08 ± 2.36
	Pearson correlation	0.683						
	*P* value	<0.001*						
	Pretarsal show, mm (mean ± SD)	3.98 ± 1.19	4.78 ± 1.30	5.23 ± 1.58	4.77 ± 1.39	5.97 ± 1.86	6.18 ± 1.71	5.44 ± 1.61
	Pearson correlation	0.361						
	*P* value	<0.001*						
	Ratio (mean ± SD)	1.11 ± 0.596	0.797 ± 0.353	0.911 ± 0.396	1.52 ± 0.715	1.04 ± 0.370	1.19 ± 0.442	1.89 ± 0.929
	Pearson correlation	0.367						
	*P* value	<0.001*						
Medial limbus	Upper lid fold, mm (mean ± SD)	2.40 ± 1.18	2.34 ± 0.89	3.19 ± 1.01	4.75 ± 1.58	4.41 ± 0.992	5.46 ± 1.39	7.49 ± 1.85
	Pearson correlation	0.777						
	*P* value	<0.001*						
	Pretarsal show, mm (mean ± SD)	3.09 ± 1.45	3.59 ± 1.47	4.14 ± 1.53	4.22 ± 1.33	5.23 ± 1.89	5.64 ± 1.73	5.47 ± 1.36
	Pearson correlation	0.533						
	*P* value	<0.001*						
	Ratio (mean ± SD)	0.857 ± 0.332	0.709 ± 0.290	0.848 ± 0.297	1.21 ± 0.445	0.936 ±0.322	1.06 ± 0.391	1.46 ± 0.530
	Pearson correlation	0.45						
	*P* value	<0.001*						
Midpupillary line	Upper lid fold, mm (mean ± SD)	3.09 ± 1.42	3.17 ± 1.26	4.13 ± 1.01	5.54 ± 1.62	5.48 ± 1.10	6.32 ± 1.13	8.10 ± 1.61
	Pearson correlation	0.781						
	*P* value	<0.001*						
	Pretarsal show, mm (mean ± SD)	2.60 ± 1.37	2.92 ± 1.32	3.56 ± 1.56	3.81 ± 1.26	4.70 ± 1.92	5.35 ± 1.80	5.38 ± 1.28
	Pearson correlation	0.606						
	*P* value	<0.001*						
	Ratio (mean ± SD)	1.50 ± 0.825	1.17 ± 0.358	1.30 ± 0.440	1.57 ± 0.563	1.32 ± 0.511	1.32 ± 0.492	1.59 ± 0.465
	Pearson correlation	0.066						
	*P* value	0.489						
Lateral limbus	Upper lid fold, mm (mean ± SD)	5.10 ± 1.82	4.95 ± 1.43	6.05 ± 1.25	7.56 ± 1.77	7.55 ± 1.26	8.21 ± 1.27	9.88 ± 1.75
	Pearson correlation	0.729						
	*P* value	<0.001*						
	Pretarsal show, mm (mean ± SD)	2.83 ± 1.27	3.01 ± 1.11	3.65 ± 1.28	4.03 ± 1.13	4.82 ± 1.67	5.45 ± 1.67	5.62 ± 1.19
	Pearson correlation	0.648						
	*P* value	<0.001*						
	Ratio (mean ± SD)	2.08 ± 0.88	1.73 ± 0.355	1.76 ± 0.433	1.99 ± 0.631	1.70 ± 0.498	1.63 ± 0.506	1.84 ± 0.508
	Pearson correlation^a^	−0.134						
	*P* value	0.159						
Lateral canthus	Upper lid fold, mm (mean ± SD)	8.27 ± 1.98	8.26 ± 1.59	9.40 ± 1.58	10.7 ± 1.92	10.8 ± 1.50	11.3 ± 1.27	12.6 ± 1.54
	Pearson correlation	0.685						
	*P* value	<0.001*						
	Pretarsal show, mm (mean ± SD)	4.23 ± 1.18	4.50 ± 0.786	4.81 ± 0.868	5.25 ± 1.22	5.88 ± 1.38	6.38 ± 1.36	6.48 ± 1.20
	Pearson correlation	0.601						
	*P* value	<0.001*						
	Ratio (mean ± SD)	2.11 ± 0.850	1.85 ± 0.267	1.97 ± 0.262	2.13 ± 0.584	1.90 ± 0.351	1.82 ± 0.340	2.03 ± 0.514
	Pearson correlation^a^	−0.052						
	*P* value	0.586						

SD, standard deviation. ^a^Negative Pearson correlation signifies an increase in variable associated with a decrease in sagittal head tilt. *Statistically significant.

**Table 4.** Spearman’s Correlation Between Periorbital Measurement & Ratio Changes and Aesthetic Rating

	Lateral canthus		Lateral limbus		Midpupillary line		Medial limbus		Punctum	
	Spearman’s correlation^a^	*P* value	Spearman’s correlation^a^	*P* value	Spearman’s correlation^a^	*P* value	Spearman’s correlation^a^	*P* value	Spearman’s correlation^a^	*P* value
Upper lid fold height	−0.317	0.001*	−0.307	0.001*	−0.361	<0.001*	−0.322	0.001*	−0.149	0.117
Pretarsal height	−0.425	<0.001*	−0.426	<0.001*	−0.415	<0.001*	−0.414	<0.001*	−0.447	<0.001*
(Upper lid fold/pretarsal) ratio	0.239	0.011*	0.182	0.054	0.081	0.398	0.003	0.974	0.151	0.113

^a^Negative Spearman’s correlation signifies higher aesthetic rating associated with a decrease in the respective variable. *Statistically significant.

## DISCUSSION

The present study is the first to demonstrate the strong influence of sagittal head tilt on periorbital attractiveness and associated topographic measurements in young female subjects. Our interest in the subject arose after we discovered the importance of the smooth youthful arcs of the lashline, upper lid crease, and brow, and the importance of the harmonious lateralization of these arc peaks from caudal to cranial which result in a more attractive and angular appearing eye.^10^ Subsequently, we applied these findings to better understand upper blepharoplasty in a series of 316 patients, discovering that improved postoperative aesthetic ratings were also significantly associated with lateralization of these upper lid arcs and creation of a relatively homogenous vertical height of the pretarsal space.^13-15^ An additional revelation occurred on close scrutiny of clinical photographs, where we noticed significant variations in periorbital appearance and the aforementioned arcs and pretarsal show due to subject head tilt. Thereafter, we noticed head tilt variations in before and after photographs in plastic surgery journals, model photographs, and patient “selfies.” It became clear that patients and models alike subconsciously prefer to tilt their head downward to improve their periorbital aesthetics.

In this study, we demonstrate that negative (ie, downward) sagittal head tilt significantly improves periorbital aesthetic ratings, as rated by 11 raters of diverse plastic surgery and nonclinical backgrounds with close inter- and intra-rater agreement. We observed that downward head tilt results in a more pronounced positive intercanthal tilt and lateralizes the brow peak closer to the lateral canthus (**Figure 9**; **Table 1**; Supplemental Video 1), which we previously demonstrated were features of more attractive female eyes.^10^ In addition, the vertical height of the pretarsal space and upper lid fold both decreased with downward head tilt, with relative maintenance of ($\frac{Upper\;Lid\;Fold}{Pretarsal}$) ratios except medially, where ratios significantly decreased with downward head tilt (**Table 3**), further contributing to an angular appearance of the eye and improved aesthetic ratings (Video 2). Though anecdotal based on our observations, we identified other interesting upper periorbital effects that varied depending on patient anatomy and early aging patterns. Some subjects demonstrated a “double lid crease,” which we believe is a consequence of upper lid fold volume loss resulting in pleating of the redundant skin envelope above the true lid crease (see Supplemental Video 2); however, if volume loss is more pronounced, the “double lid crease” can disappear and instead will reveal excess pretarsal show and medial hollowing, resulting in an unesthetic reversal of ($\frac{Upper\;Lid\;Fold}{Pretarsal}$) ratios (see Supplemental Video 3). In these subjects, negative sagittal head tilt appears to recruit skin into the lid crease through levator activation, helps decrease apparent pretarsal show excess, helps conceal medial upper lid fold hollowing and also helps smoothen and lateralize the lid crease arc. (Video 3). In contrast, in eyes with relatively small pretarsal spaces and low set brows, raters on average rated −5° or −10° views as most attractive, and −15° less attractive (see Supplemental Videos 4, 5). We hypothesize that this is due to opacification of the pretarsal space and excess downward angulation of the medial brow which can result in the eyes looking too deep-set or result in a menacing appearance (Video 4).

A notable exception to the above pattern was in eyes with lower eyelid bags (see Supplemental Videos 5, 6). While downward head tilt was correlated with an overall improvement in perceived attractiveness, eyes with lower lid bags were rated as most attractive at neutral head tilt in both 3-view and 7-view ratings (**Figures 5, 7**). In the 3-view ratings (n = 24 eyes), negative vector eye attractiveness ratings appeared to plateau at 0° (**Figure 4**); however, no significant difference was observed in the 7-view rating (n = 10 eyes), likely due to the 7-view component of the study being underpowered. Our clinical interpretation of these findings is that downward head tilt aesthetically not only improves upper periorbital features but also results in increased demarcation/shadowing along the eyelid-cheek junction which is much more noticeable in eyes with lower eyelid bags (which are more common in negative vector eyes). If eyelid-cheek junction demarcation is significant enough, it can negate or overwhelm the upper periorbital aesthetic gains obtained with downward head tilt (Video 5).

Another noteworthy observation was the effect of sagittal head tilt on MRD1 and MRD2. Along the tested head tilt ranges of −15° to +15°, MRD1 remained consistent; however, MRD2 (and lower scleral show) increased with negative head tilt (**Figure 9**). Anecdotally, increased scleral show with downward head tilt was more apparent in negative vector eyes. Clinically, we have noticed lower blepharoplasty before and after photographs in several scientific manuscripts claiming to have “maintained” lower lid margin position, yet with the patients clearly tilting their heads backward in the postoperative image. Therefore, maintenance of lower lid margin position can only be assessed if similar sagittal head tilt is maintained between photographs. We thus propose a simplified means to assess and control for sagittal head tilt by including the tragus in periorbital clinical photographs (Video 6).

In behavioral psychology, downward sagittal head tilt while maintaining forward gaze and neutral expression has been shown to increase the perception of subject dominance.^16,17^ Interestingly, however, the relationship between perceived dominance, attractiveness, and masculinity and femininity has been shown to be quite complex and different among male and female subjects but consistent regardless if the rater was of the same or opposite gender.^17,18^ Nonetheless, female faces have been shown to exhibit an increased perception of femininity and attractiveness with downward head tilt.^17,19,20^ However, these studies did not control for the influence of other facial features, as whole facial images were assessed in these studies. Furthermore, these studies were designed by behavioral psychologists; in contrast, plastic surgeons are more focused on the subtle nuances of curves, surface topography, and light highlight/shadow effects. In the periorbital area, seemingly miniscule differences in head tilt can impart a profound effect on eye aesthetics. Our goal was to specifically assess the influence of sagittal head tilt on the perception of periorbital attractiveness of female subjects for the goal of accurate representation and comparison of preintervention and postintervention plastic surgery clinical photography.

There are several limitations to this present study. We used young female subjects as this was a pilot study/demonstration of concept which still successfully assessed how sagittal head tilt affects periorbital appearance. In older patients, it is possible that these effects are more pronounced as patients often present with ptosis, dermatochalasis, and brow ptosis. Furthermore, the aged eye is even more sensitive to light/shadow effects due to greater skeletonization at the interface between the periorbital and midfacial subunits. Nonetheless, many of the young female eyes assessed in this study demonstrated features commonly seen in the aged eye including varying severity of medial upper lid fold hollowing and lower eyelid orbital fat herniation; therefore, our findings carry clinical relevance to patients presenting for blepharoplasty. Additionally, we only obtained 2-dimensional (2D) photographs. Three-dimensional (3D) imaging has significantly improved our ability to assess facial contour and volume. 3D photographs can be used to rotate and adjust head position. However, this cannot simulate the effect of neck flexion/extension and change in gaze direction, as eye gaze averted from observer (or camera lens) has been shown to result in the perception of shame and submissiveness.^16,21-23^ Furthermore, 3D photographs do not capture the effect of upper lid elevators and lower lid retractor musculature with sagittal head tilt and tend to produce significant rendering artifact along the lashline and cornea; therefore, 3D photography was not used. This study may have been underpowered to demonstrate other associations and would have likely benefited from a greater number of eyes with lower lid bags and a negative vector. Regarding photography methods, it is possible that there were slight errors in the accuracy of obtaining images at the correct head tilt. We attempted to minimize this by using a single observer. Nonetheless, the reported trends in periorbital appearance with head tilt would not be affected. Earlier in the study, we only obtained photographs in the first 7 subjects at −15°, 0°, and +15° head tilt. We subsequently noticed how profound the periorbital changes were at these increments, leading us to expand our photography methods to include additional 5° increments. We decided to obtain 2 sets of ratings where raters were first presented with 3 views and then with 7 views. The reason for this is that the 3-view rating initially served as a form of rater training and we were additionally concerned that raters may be overwhelmed by the 7-view rating with too many choices.^24^ This study could have potentially benefited from a greater number of evaluators, including a greater number of plastic surgeon evaluators. Nonetheless, our inter-rater and intra-rater reliability calculations of >0.9 are indicative of excellent agreement among and consistency of our evaluators, respectively, despite the heterogeneity of the evaluator’s educational backgrounds—we believe this adds further strength to our study’s conclusions. Further supporting this point, cognitive research studies have demonstrated that opinions of facial attractiveness are highly consistent among individuals despite differences in social, economic, and ethnic backgrounds.^18,25,26^ Future studies may aim at investigating the effects of sagittal head tilt in a greater number of subjects, different age ranges, males, patients with ptosis, and among different subject ethnicities.

## CONCLUSIONS

Overall, negative sagittal head tilt significantly improves periorbital aesthetics; however, in the presence of lower eyelid bags, this also increases demarcation of the eyelid cheek junction which may be aesthetically detrimental. Controlling for sagittal head tilt is critical to reliably compare preoperative and postoperative clinical photographs.

## Supplementary Material

ojab043_suppl_Supplementary_Appendix_A

ojab043_suppl_Supplementary_Appendix_B

ojab043_suppl_Supplementary_Video_1

ojab043_suppl_Supplementary_Video_2

ojab043_suppl_Supplementary_Video_3

ojab043_suppl_Supplementary_Video_4

ojab043_suppl_Supplementary_Video_5

ojab043_suppl_Supplementary_Video_6
