# Supplementary material for: The Influence of Sagittal Head Tilt on Periorbital Appearance: Implications for Clinical Photography and the Evaluation of Postoperative Results
Source: Aesthet Surg J Open Forum. 2021 Oct 30;4:ojab043. doi: 10.1093/asjof/ojab043 (PMC8830303; doi:10.1093/asjof/ojab043)

For each slide (7 images per slide):

Rank the following cropped eye images from 1 – 7

1 = Most Attractive

7 = Least Attractive

We suggest you start by ranking the <u>most</u> & <u>least</u> attractive eye images first

Please type the numeric score in the associated blue text box <u>below</u> each image

An individual eye image may possess features that are both *more* and *less* attractive than another eye image – however, **please rank each image based on your OVERALL impression of attractiveness**

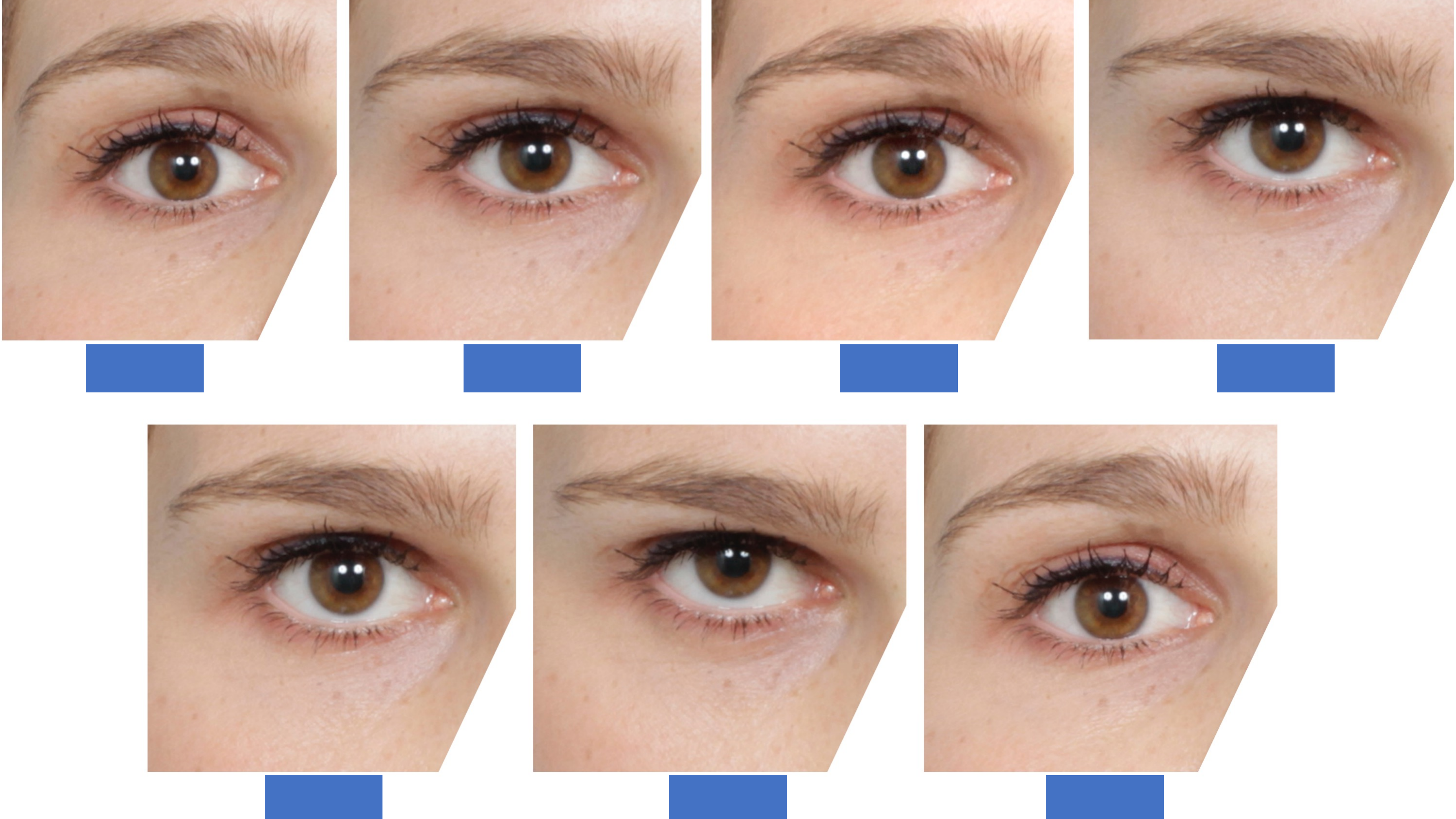

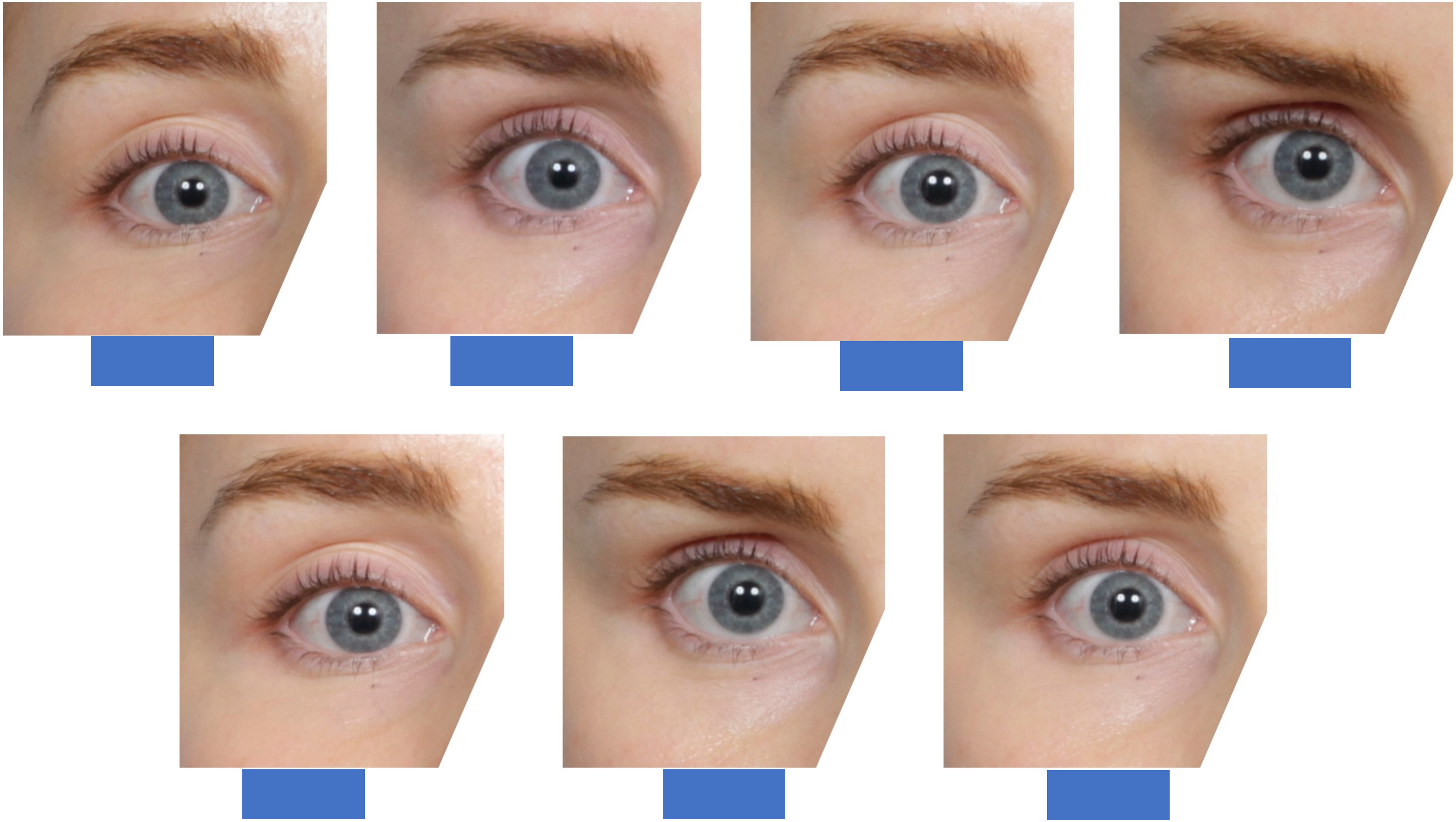

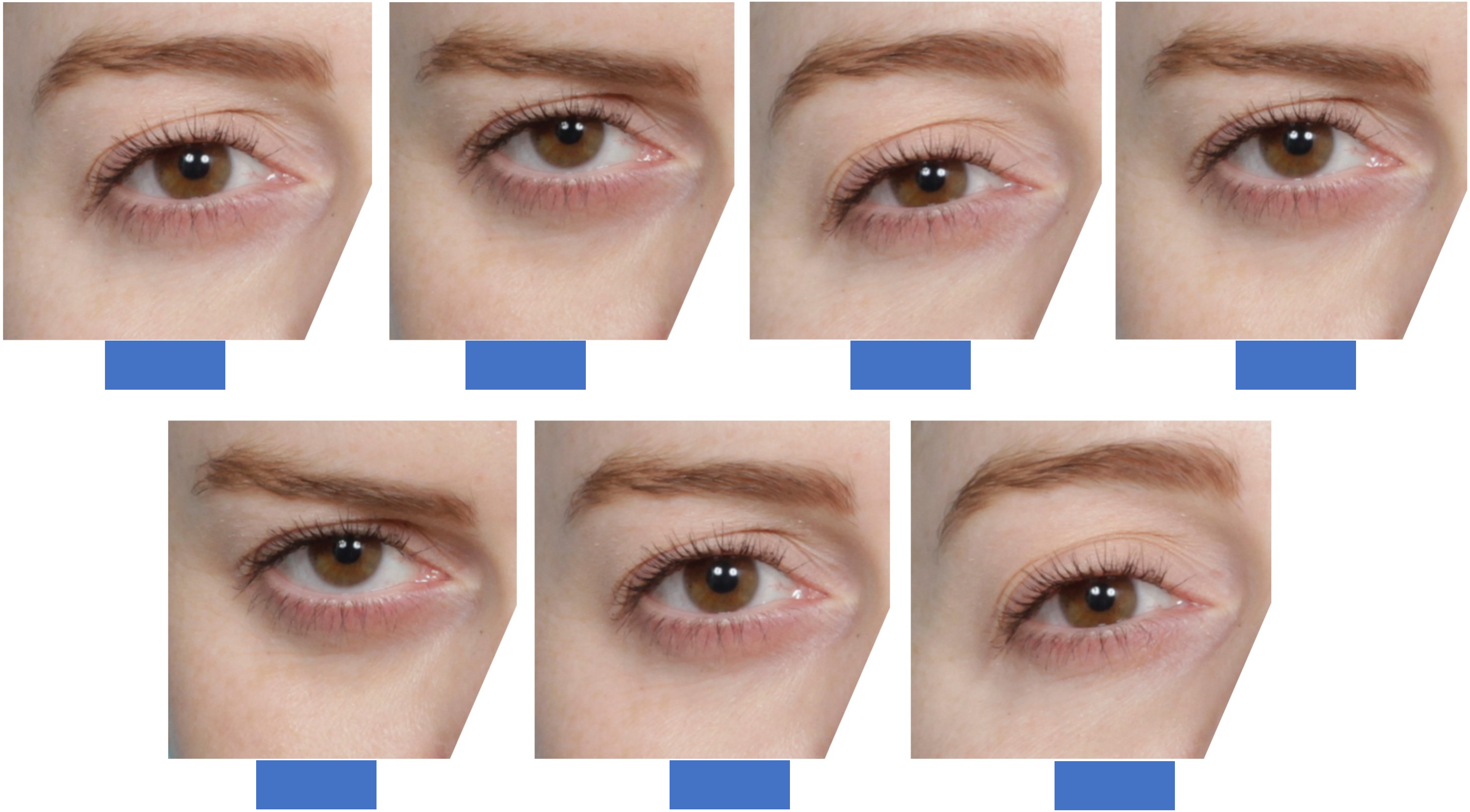

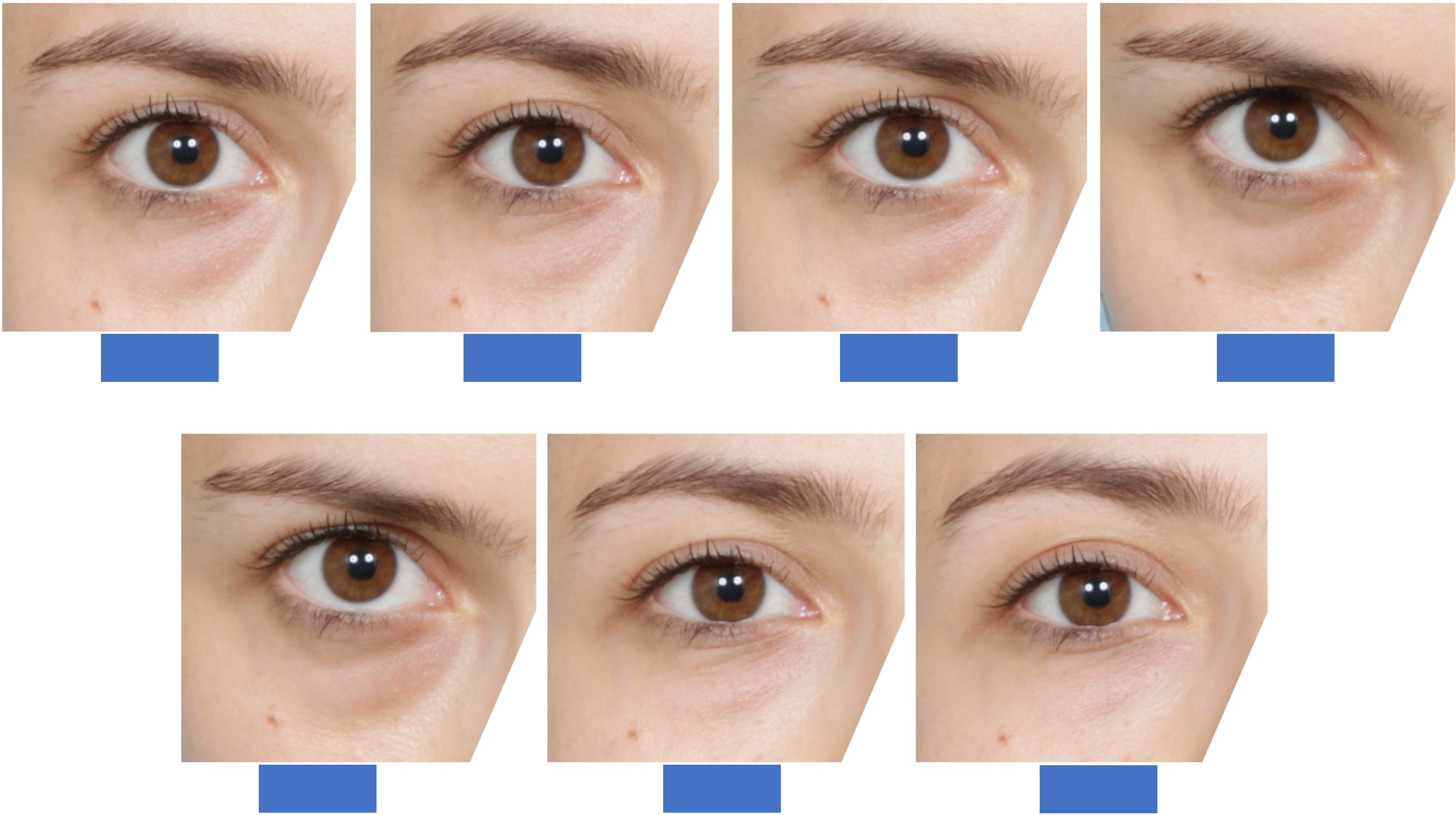

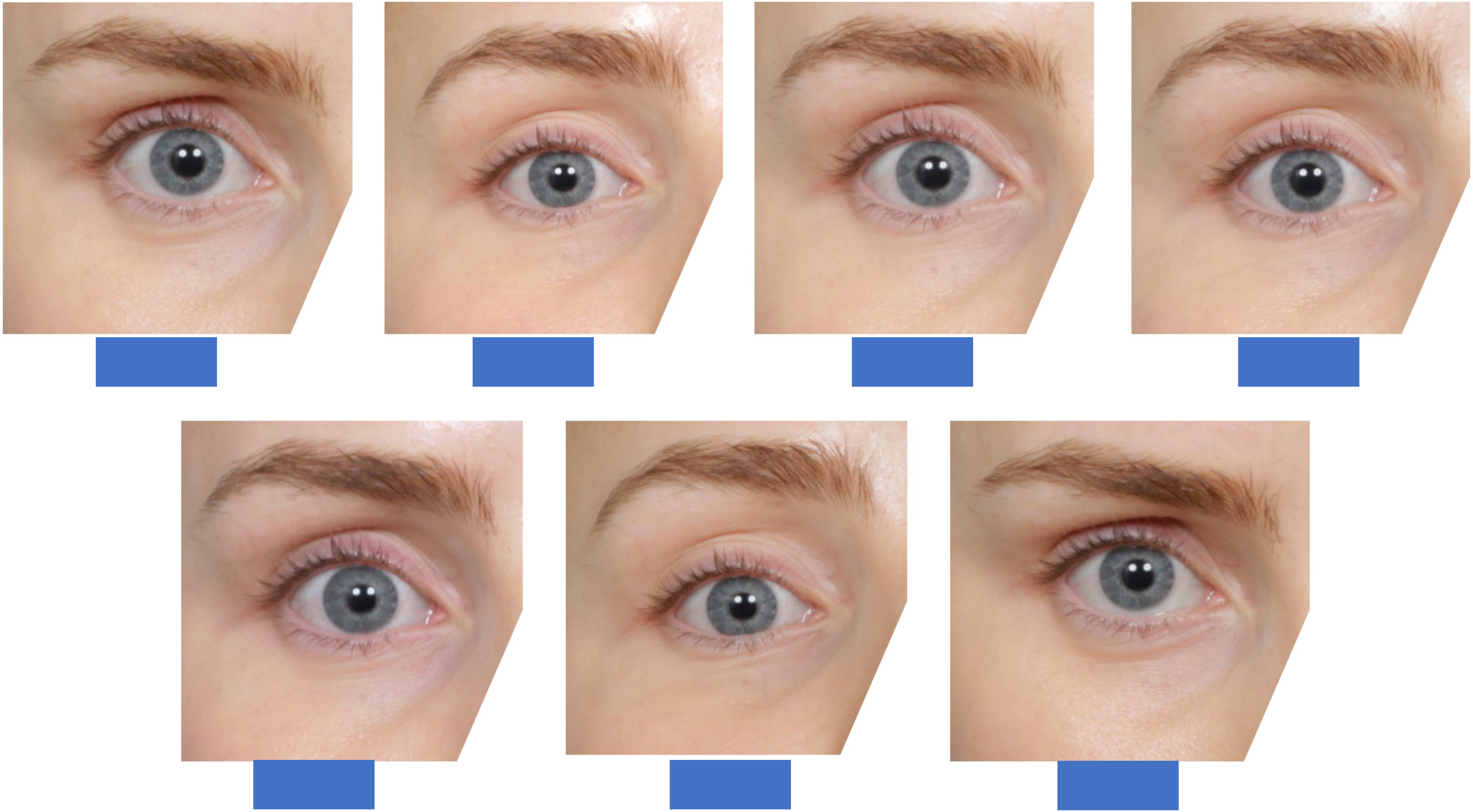

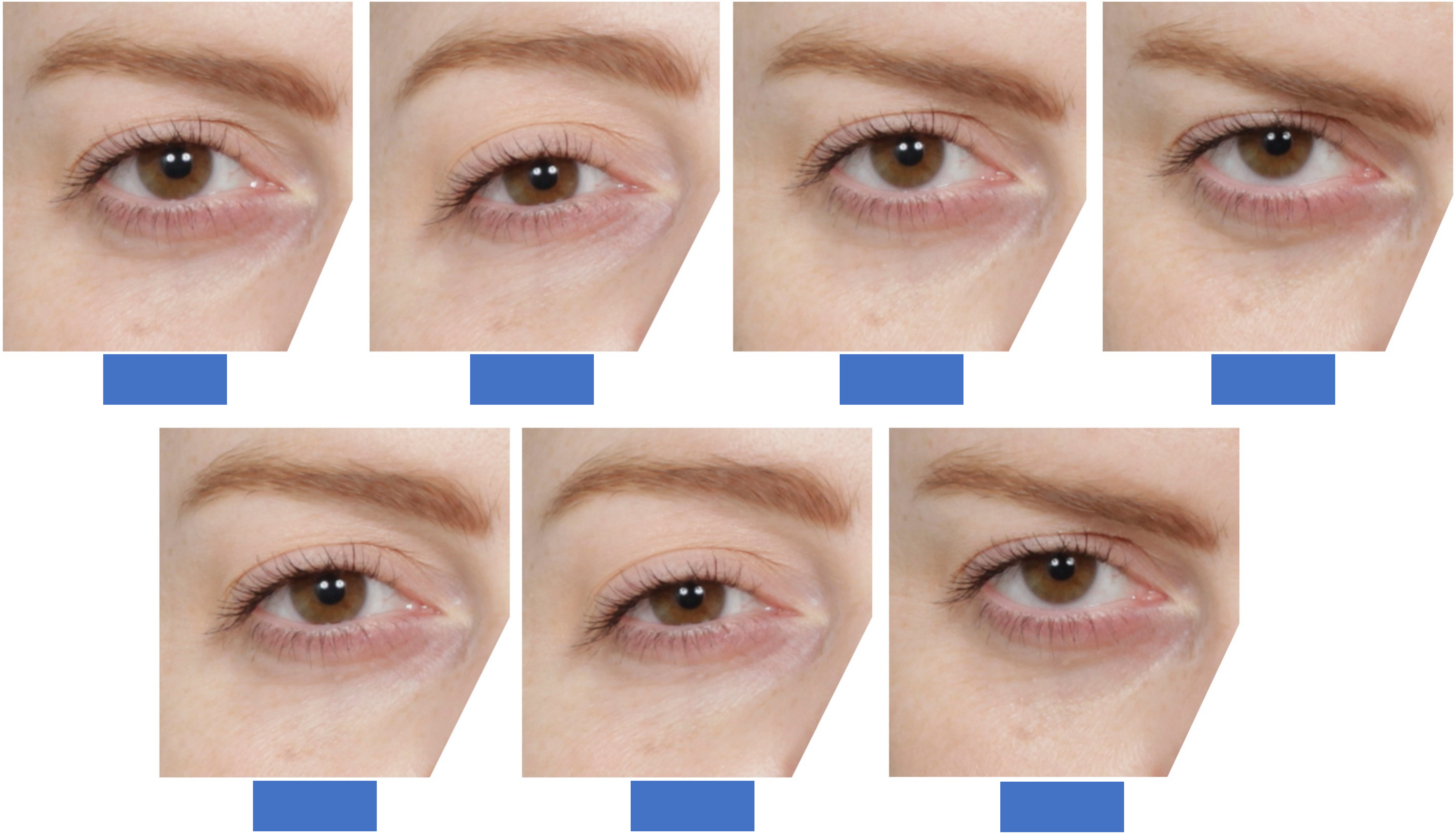

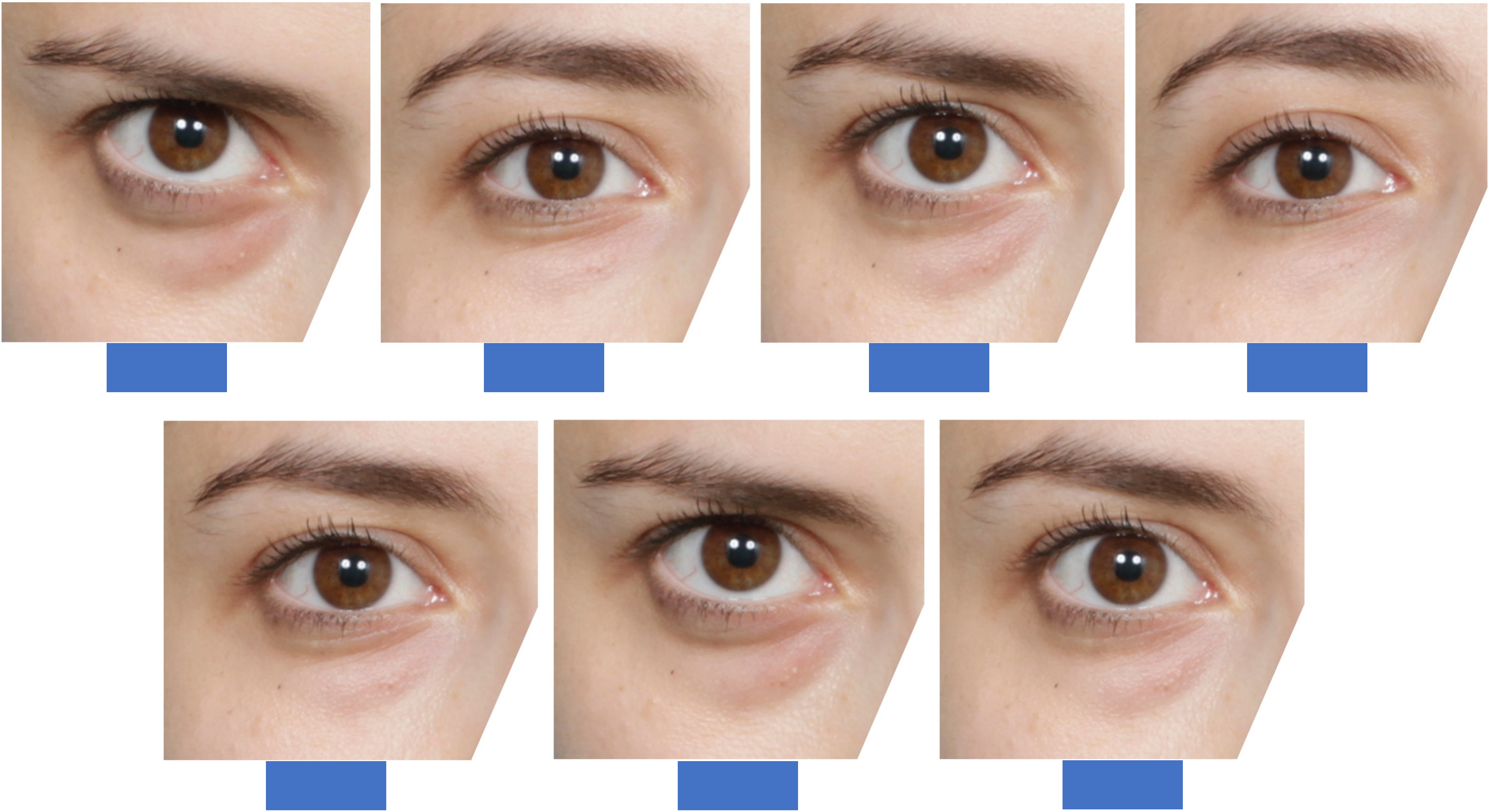

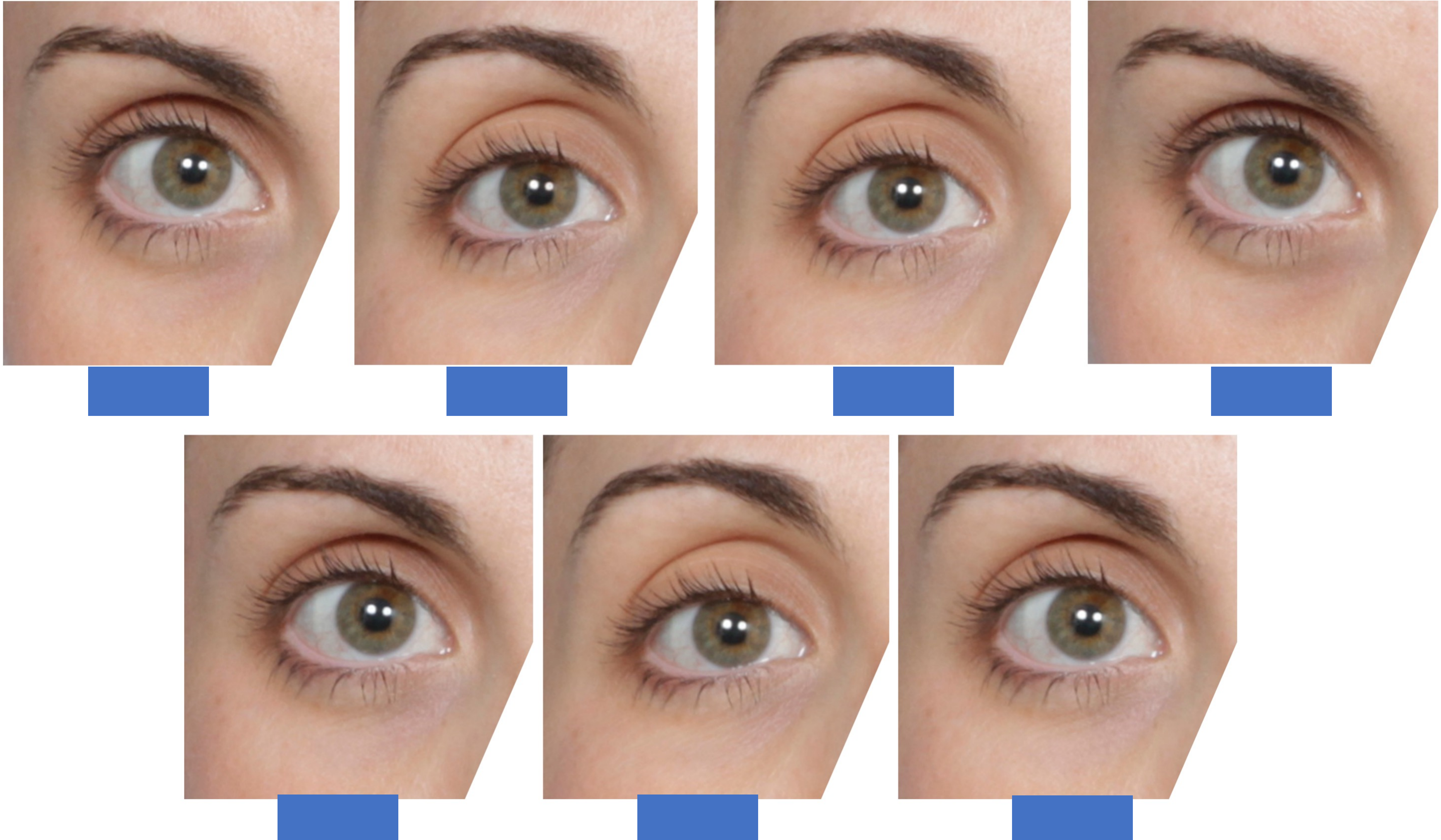

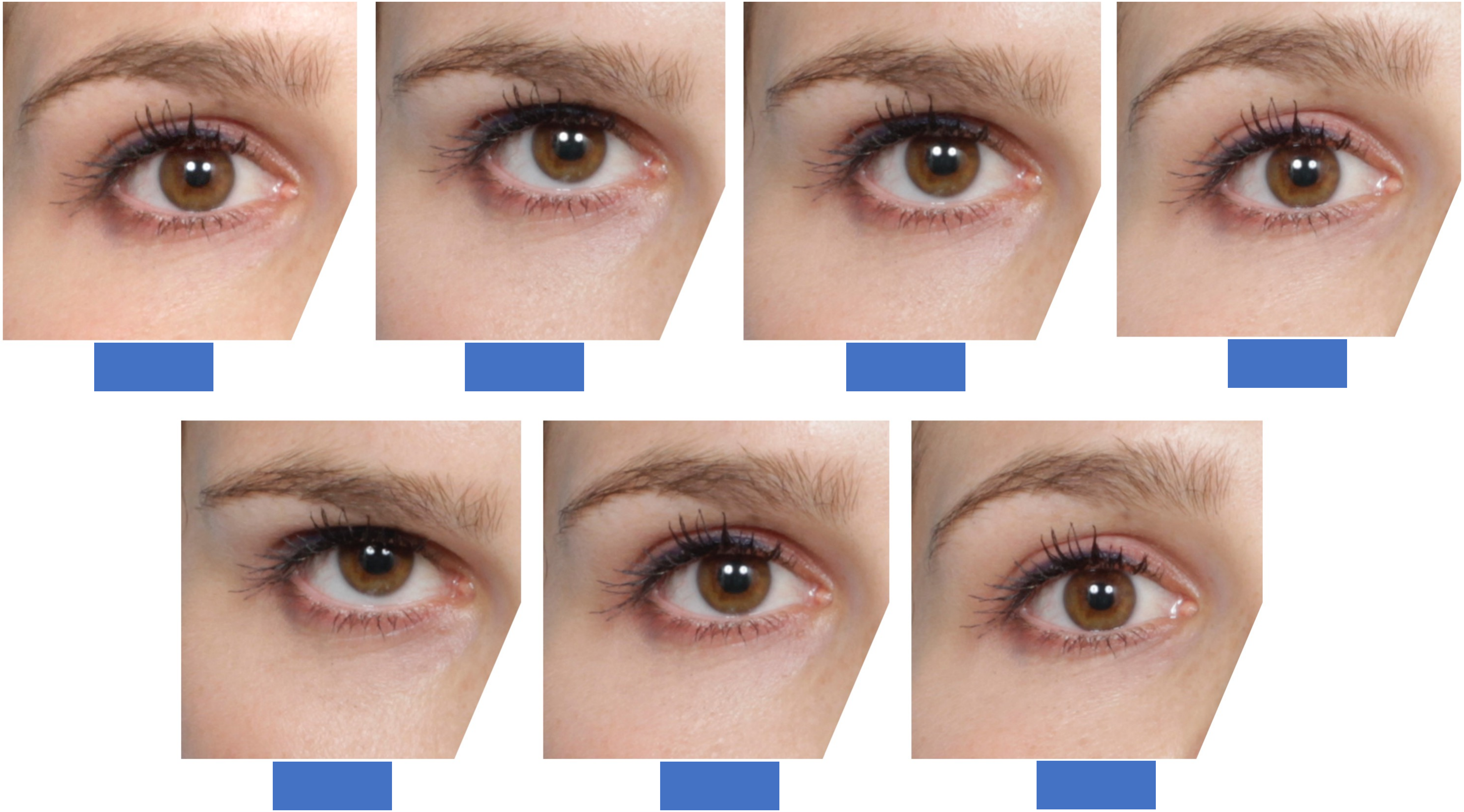

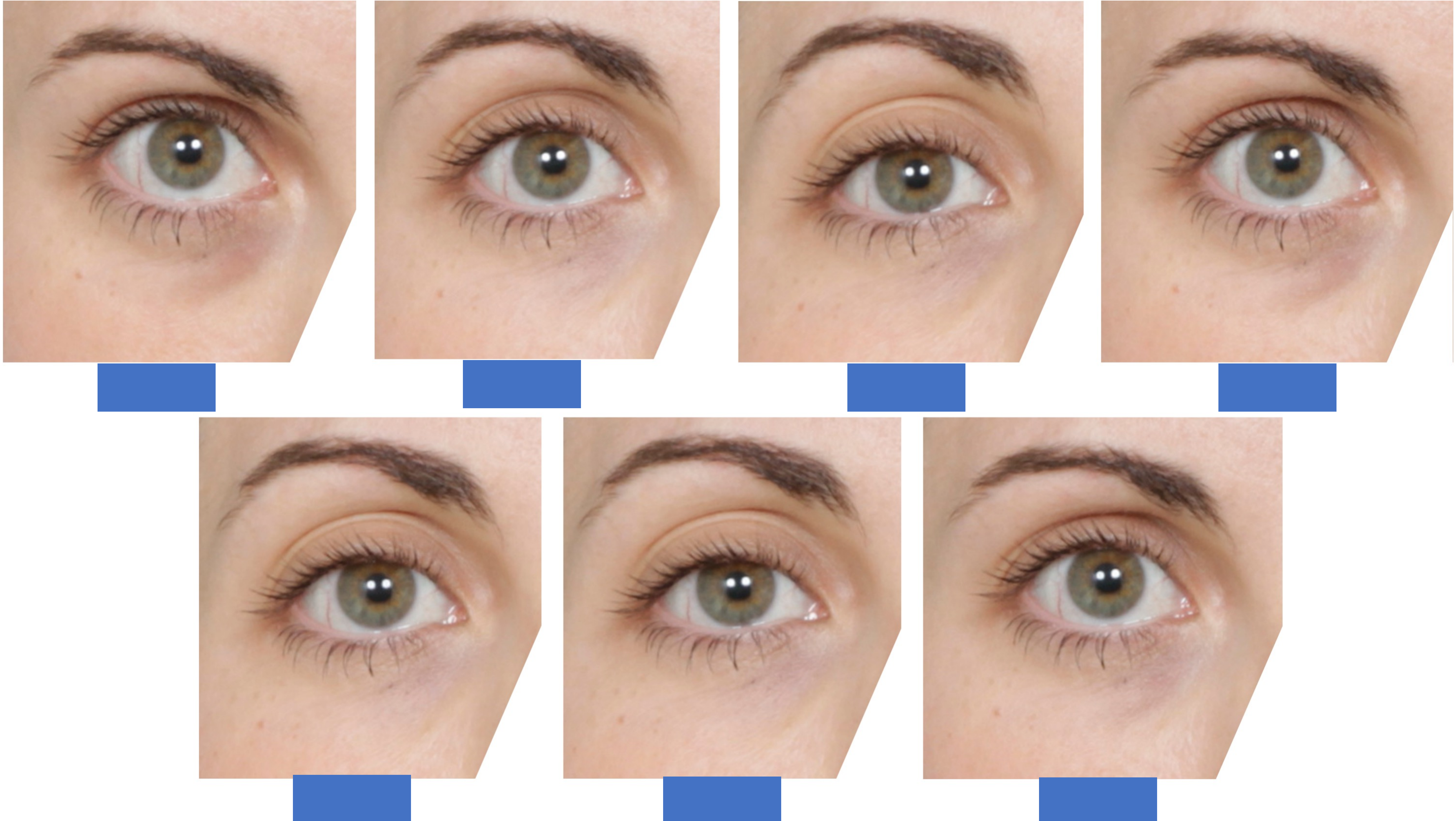

Supplement: ojab043_suppl_Supplementary_Appendix_B [file ojab043_suppl_Supplementary_Appendix_B.pdf]
